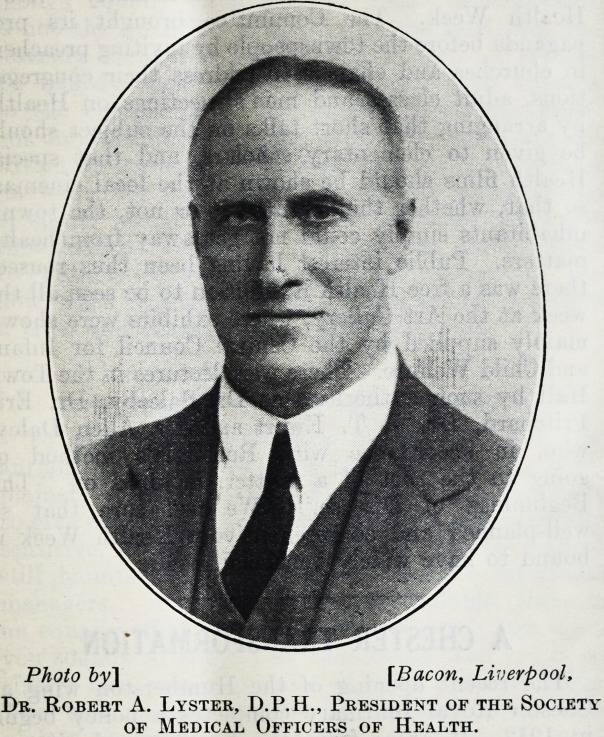# Public Health Administration: Interview with Dr. Lyster

**Published:** 1924-12

**Authors:** 


### PUBLIC HEALTH ADMINISTRATION.

### INTERVIEW WITH THE PRESIDENT OF THE SOCIETY OF MEDICAL OFFICERS OF HEALTH.

The Society of Medical Officers of Health possesses in its president, Dr. R. A. Lyster, who is Medical Officer for Hampshire and resides at Winchester, a man who is keenly concerned for the future of public health administration in this country, and is determined, so far as lies in his power, to inspire in the members of the society something of his enthusiasm for efficiency and of his will to move along the lines of ordered progress. Dr. Lyster very courteously responded to our request for an interview, and gave expression of his views on many subjects to which

*Photo by*] [*Bacon, Liverpool,*

Dr. Robert A. Lyster, D.P.H., President of the Society of Medical Officers of Health.

limitations of space make it somewhat difficult to do justice.

### Idealism or Cant.

At the outset Dr. Lyster said that he thought that there was at the present moment a great opportunity to be seized. A vein of idealism was running through the present disturbed life of Europe. It would be a fine thing to seize this opportunity and to direct the public enthusiasm for progress into proper channels. At the same time he uttered a warning against the possibility in various forms of cant masquerading as ideals. He struck this note in his recent presidential address, in which, among other things, he challenged the abuse in practice of such phrases as "the undermining of parental responsibility," in connection with the relief of suffering and the promotion of health, and "the maintenance of the great voluntary principle" as applied to services that are paid.

### The Voluntary Worker.

Invited to develop his views with regard to the position of voluntary workers in public health administration, Dr. Lyster said, without qualification, that it was in the sphere of administration of the legal responsibilities of local authorities that he thought the voluntary worker made the most mistakes. Administration, indeed, was not at all the sphere for the voluntary worker. He felt that where public funds of any kind were involved their administration should be directed by the local elected bodies who had responsible and properly trained officers, and were themselves responsible to the electorate for the proper use of the funds entrusted to them. Asked whether he went so far as to wish to get rid altogether of voluntary organisations, Dr. Lyster explained that where funds were definitely contributed by individual members of the public for maintaining a voluntary organisation, he saw no reason why the organisation should not have a free hand with the funds so contributed. If they muddled away the money, or if they failed to employ adequately trained and disciplined workers, the charitable public would have the matter in their own hands. When, however, it came to supporting voluntary bodies with grants from the rates, or from the public exchequer, Dr. Lyster felt very strongly that these bodies should come under the definite control of the local authority. He claimed that in Hampshire every encouragement is given to genuine voluntary workers, and their services are used in every branch of the work with great success.

### District Nursing Associations.

In an imperfect world where facts have to be accepted until there is greater enlightenment, Dr. Lyster does not carry his theories to the extent of refusing support to bodies that are working in harmonious relation with the local authority. In his own county—Hampshire—he has himself constantly recommended to the Council to make grants to struggling district nursing associations. It is curious that these recommendations have been opposed in quarters where support for the voluntary principle might have been supposed to have been particularly active.

### The Best Voluntary Worker.

As to the best voluntary worker, let us give Dr. Lyster's own words in his presidential address: "It is a curious mentality which puts a halo round self-appointed members of so-called 'voluntary associations' and their (usually) unqualified officers. The best voluntary worker is the popularly elected representative of the public upon the various local authorities. His services are purely voluntary, and every medical officer of health is the expert administrative officer of a body of genuine 'voluntary workers' who are in their turn responsible to the public."

### Future Developments.

In regard to the co-ordination and development of public medical services to which reference is made from time to time by Ministers and others Dr. Lyster is a "whole-hogger." Abolition of the Poor Law would be an essential first step to the linking up of the whole of the public medical services in a county area under the Health Committee of the County Council. The sanitary administration of the municipal boroughs and larger urban districts would be excluded from this control, bur these areas would have to find their place in a proper scheme for the larger area and be definitely related to the activity of the smaller authorities and the county as a whole.

WHOLE-TIME MEDICAL OFFICERS.

As a useful step in the linking up of public medical services Dr. Lyster is pursuing a plan whereby, as vacancies occur in small areas among part-time medical officers of health, these are gradually filled up by whole-time medical officers working in conjunction with the County Council. [We have referred in an earlier article to something of this kind which is working in Essex with apparently most successful results, although in Essex the definite grouping of areas is usually arranged as the first step.]

THE PATH OF PROGRESS.

With regard to the views of the President of the Society of Medical Officers of Health on the training of health officers, the protection of the public from faddists, the need for organised public propaganda, and as to research work, we may refer our readers to the address printed in the November number of *Public Health.* Dr. Lyster concluded his talk with us with a reiteration of what he had already said as to the need for courage in fighting the forces which threaten to damage or destroy the human race. He is confident, however, that we are winning.

## Figures and Tables

**Figure f1:**